# Use of race, ethnicity, and ancestry data in health research

**DOI:** 10.1371/journal.pgph.0001060

**Published:** 2022-09-15

**Authors:** Clara Lu, Rabeeyah Ahmed, Amel Lamri, Sonia S. Anand

**Affiliations:** 1 Department of Medicine, McMaster University, Hamilton, Ontario, Canada; 2 Arts and Science Program, McMaster University, Hamilton, Ontario, Canada; 3 Department of Health Research Methods, Evidence & Impact, McMaster University, Hamilton, Ontario, Canada; The University of British Columbia, CANADA

## Abstract

Race, ethnicity, and ancestry are common classification variables used in health research. However, there has been no formal agreement on the definitions of these terms, resulting in misuse, confusion, and a lack of clarity surrounding these concepts for researchers and their readers. This article examines past and current understandings of race, ethnicity, and ancestry in research, identifies the distinctions between these terms, examines the reliability of these terms, and provides researchers with guidance on how to use these terms. Although race, ethnicity, and ancestry are often treated synonymously, they should be considered as distinct terms in the context of health research. Researchers should carefully consider which term is most appropriate for their study, define and use the terms consistently, and consider how their classification may be used in future research by others. The classification should be self-reported rather than assigned by an observer wherever possible.

## Introduction

Over the past two decades, ethnicity and race-based health research has accelerated [1, 2]. In parallel, an increasing number of population genomics studies are being conducted in which the term ancestry is often used. Currently there is confusion regarding which is the optimal term to use in health research–ethnicity, race, or ancestry, partly because they lack standard definitions. Conducting such research necessitates being aware of the differences between the concepts of ethnicity, race, and ancestry, and requires that clear definitions of these terms be made explicit by the researcher [3]. This confusion is fuelled by three contemporary perspectives: i. the contention that human populations are more alike than different, ii. genome-wide and whole genome sequencing technology which generates ancestral genetic information, and iii. the societal reckoning with structural racism in healthcare provision and practices. These perspectives justify a re-examination of the concepts and terms of ethnicity, race, and ancestry. This article will review evidence that informs our understanding of these terms, identify gaps in our understanding, and provide direction to researchers regarding the use of these terms in health research.

## Methods

We conducted literature searches in PubMed to identify published manuscripts in the English language for i. ethnicity and race and health research, ii. ethnicity and race with ancestry and genetics, and iii. ethnicity and religion. All search strategies are described in S1 Table. Key articles were then selected and reviewed in depth, and others identified through a review of the citations to generate this narrative review [4].

### 1. Use of “race” and “ethnicity” terminology in health research

Race and ethnicity are often used as classification variables in health research. However, the terms are frequently misused and invoked inconsistently [5, 6]. Race is a socio-political construct that divides people into groups based on perceived physical differences [7, 8]. For more than two centuries, this classification has widely influenced medical education and led to racial discrimination in healthcare [9]. The persistent use of race categories in medicine has had two significant impacts, leading to controversy regarding the ongoing utility of this classification in health research. On one hand, the use of the term race has perpetuated the incorrect belief that race categories reflect biological “including genetic” differences, which may reinforce harmful stereotypes and perpetuate “race science” [3, 10–14]. This calls into question whether measuring race is an effective way for health researchers to analyze differences between various groups and whether overall findings from studies that assess race can be generalized to the broader population. On the other hand, race indicators can help identify and address inequalities arising from discrimination [8, 15, 16]. As categorization of human populations by race fuels racism, and people affected by racism are referred to as “racialized,” the reporting of racial differences may illuminate the powerful effects of social inequalities on health and healthcare. The use of the term ‘race’ therefore may be used as a proxy to account for social inequalities related to racism and its socio-political disparities. For the practical benefit of monitoring health inequities that arise from racism, some argue that we should continue to measure “race” [17]. This however is heavily dominated by the American perspective, and is not common in Europe, where the term race is rarely used [18]. However the European Union Commission has recently provided a framework for disaggregating data by ethnicity and race to quantify discrimination and inequity, with the goal of benefiting the groups they describe [19].

As race is widely acknowledged to be a social rather than a biological construct, the resulting challenges associated with racial classification and data collection have led some researchers to prefer the term “ethnicity.” Ethnicity is a construct that encompasses common cultural characteristics including language, religion, dietary practices, and nationality; it *may* also reflect common ancestry or geographic origin [8, 15, 19]. These characteristics create a sense of collective identity that is often carried forward between generations [20]. Ethnicity categories can be used to design “culturally appropriate health services” and investigate clinical-biological differences in risk factors for diseases and responses to therapies [8, 21]. Some religious groups for example Jewish populations can also be included as ethnicity categories, as this can be the dominant identity for some individuals more so than country of origin or skin colour in the case of race-based classifications. To account for various cultural and socioeconomic factors that may influence population health outcomes, some researchers may choose to define ethnicity in terms of religious affiliation and categorize the Jewish population as a distinct ethnic group [22].

Although some researchers advocate for using the term ethnicity instead of race, using the term ethnicity alone without a precise definition can also cause confusion [23]. Ethnicity is a complex, multidimensional construct that may change over time, and this contributes to its within-group heterogeneity. While the construct of race relies on perceived physical characteristics, ethnicity also captures elements of an individual’s identity beyond the physical, as previously described. As a result, the use of each term is subject to various interpretations by researchers, study participants, and readers [23]. Researchers have noted that the racial groups traditionally classified by the United States (US) Census Bureau–such as Black or African American, Asian, American Indian or Alaska Native, Native Hawaiian or Other Pacific Islander, and White–can themselves include multiple ethnic groups. For example, Black, White, and Asian races can all include individuals belonging to Hispanic ethnicity [23]. Recently, calls for a re-evaluation of this antiquated definition and use of race categories by the US Government have been made [20].

Finally, there has been no standard agreement on the distinction between the terms ethnicity and race in the context of research [3, 7, 24]. No formal consensus has been reached regarding which term is preferable to the other, and in which circumstances. Despite substantial literature emphasizing the consequences of using these terms interchangeably, in practice, the two terms are often treated as synonyms [5, 24–26]. As such, it is essential for researchers to clearly define their chosen terms.

### 2. Implications of misuse and confusion of terms

A 2011 systematic review highlighted studies that have misused and confused the terms race, ethnicity, and ancestry, and noted that researchers often did not justify why recording these variables was necessary [27]. Many studies featured in the review did not define these terms in the context of their research [28–33]. In addition to inconsistencies within individual manuscripts, authors often used varying terminology to describe the same concepts. For instance, in manuscripts focused on genetics, authors variably described their findings in terms of ancestry [29], race [30], and ethnicity [31]. In some publications, the descriptors used in the main article were different from those in the supplementary text [31, 33]. Finally, some authors introduced multiple terms without defining or explaining whether each of the terms are distinct or synonymous in the context of their study [34, 35]. It is unlikely that comparing populations in this manner is meaningful and this ultimately causes substantial confusion amongst readers [35].

When researchers fail to define “race” and “ethnicity” in their research, they reinforce the idea that racial and ethnic classifications are unchanging and well-defined [36]. This also fuels the assumption that biological differences may exist when racial differences are reported regarding differences in medication efficacy, for example [37], thereby perpetuating the belief that racial differences reflect biological differences. While some biological-race examples appear to be firmly ensconced in the practice of medicine, a recent report demonstrated that risk models that do not include race can be more precise and can replace such race-based formulae [38].

The internalization of racial stereotypes by healthcare professionals can lead to disparities in healthcare delivery for non-white people. Physician’s referral bias for cardiac catheterization based on gender and race after assessing actors describing anginal pain. was well illustrated by Schulman et al. [39]. The observation that using race as a descriptor in medical histories can invoke such bias has led to calls for the removal of this characteristic from case presentations [40]. In contrast, when ethnicity or race is not collected, or when under-reporting occurs in situations where individuals want to avoid stigmatization, this can lead to under-recognition of ethnic and racial health disparities and discrimination when they do exist [41].

Precise and accurate assessments of race and ethnicity are important if they are to be used in health services research. The race-and ethnicity-specific data that emerge from clinical studies are often used by governments, private and public institutions, and businesses to create health interventions and to distribute healthcare resources based on priority. Since race and ethnicity are widely used in health services research, consistent terminology and measurements are essential for identifying health services gaps that may be race-and/or ethnicity-specific [23]. However, ethnicity and/or race data can be variably collected in electronic health records (EHR) and are not routinely collected in paper-based records in some countries like Canada. This lack of standardized ethnicity data has prompted the development of surrogate measures, such as unique surname analysis to assign ethnicity for some ethnic groups like South Asian and Chinese people [42, 43]. This practice, however, cannot be readily applied to most ethnic groups. Furthermore, some analyses of EHR have identified serious gaps in completion of the race and ethnicity fields when these characteristics require “assignment” by an observer, rather than by patient self-report. The race and ethnicity categories available, and whether these fields are assigned or self-reported, also need to be carefully considered as outlined below.

#### Discrepancies between self-reported and observer-classified race or ethnicity

The validity of race and ethnicity classification may depend on whether it is *self-reported* by a research participant or patient or *assigned* by a research assistant or healthcare worker (i.e. observer-classified). The relationship between self-reported and observer-classified race and ethnicity has been described in several studies using population-based survey data from the US and Latin America [16, 44–48]. Some of these surveys transitioned from observer-recorded to self-reported race in response to federal guidelines, for example from the US Office of Management and Budget in 1997 [47]. Observers within these studies included public health researchers, government officials, census enumerators, medical examiners, hospital or clinic personnel, and next of kin [49–51] with wide variations in observer training on race and ethnicity classification [16, 52–54]. An analysis of the US health survey data from the Behavioral Risk Factor Surveillance System found that agreement between self- and observer-identified race varied across racial and ethnic groups. Higher agreement rates existed among self-identified Black (96% agreement) and White (98% agreement) participants, with lower agreement rates among non-Black minority groups (35% agreement among Native Hawaiian and other Pacific Island participants) [45]. An analysis of US Veterans Affairs healthcare users also showed high agreement rates among White (98%) and Black (94%) racial groups and low agreement rates among non-Black minority groups [47], with similar patterns demonstrated across studies [52, 55–57]. These trends are not unique to the US. An analysis from the Project on Ethnicity and Race in Latin America reported that only 61% of classifications by interviewers matched those of respondents [46] while an overall agreement rate of 75% between participants and observers was reported in the Brazilian Social Survey [44]. Again, in Latin American populations, high agreement rates were found for White respondents but lower reliability existed between self and observer classification for minority respondents [16, 44, 48]. “Passing” is a commonly described phenomenon in which an individual assumes the identity of a different racial or ethnic group to increase socioeconomic status [53, 58]. Socially-assigned race refers to the concept of an observer making assumptions of an individual based on physical appearance in particular skin colour [45]. Being socially-assigned as “White” is associated with higher health status, even among those who self-identify as non-White [45]. Other factors beyond skin colour can affect observer classification of race and ethnicity. In addition to an observer’s interpretation of both visual cues (e.g. skin tone or hair texture), auditory cues (e.g. accent or vernacular) may affect their assignment of race or ethnicity [53, 59].

Discrepancies between self-reported and observer classification of race and ethnicity can have substantial implications on health research findings. For example, observer-assigned compared to self-identification of race has led to underestimations of infant mortality and cancer incidence of Native Americans [51]. On the other hand, individuals who are classified by others as White, irrespective of their self-identified race, are more likely to report their health status as being very good or excellent compared to individuals who are not socially-assigned as White [45]. It is plausible that being classified by others as White could positively bias observer assessments of other health-related outcomes, such as patient health literacy and adherence to interventions, thereby reinforcing racial stereotypes and hierarchies [6, 48].

For all of the above reasons, we conclude that self-reported identity is preferred over observer classification [17]. However we contend that in some circumstances, measuring both self-reported and observer assigned race and ethnicity may be informative. For example, observer classification of perceived race and ethnicity may illuminate the impacts of discrimination within and outside healthcare, particularly when outcomes vary substantially between self and observer classification [44, 56]. Further research is needed to discern the impact of different race and ethnicity classification methods on findings within health research [45].

#### Reliability of self-reported ethnicity or race over time

Evidence that self-reported ethnicity and race can change over time challenges the assumption that these variables remain fixed across the life span [51, 53, 56, 58, 60]. Census studies demonstrating limited reliability of ethnicity and race data date back to 1974, when a third of the US population reported a different racial or ethnic status one year after their initial interview [61]. A more recent analysis of US census data found that 6.1% of respondents–approximately 9.8 million people–reported a different race in 2010 compared to 2000 [58]. This phenomenon of racial fluidity appears most pronounced among minority and multiracial populations. Saperstein et al. describe such phenomena as “emblematic of how racial fluidity interacts with racial inequality,” with the reporting of race closely tied to historical and ongoing racial discrimination within society [53, 62].

Only 40% of people who reported multiple races in the 2000 US Census subsequently reported multiracial status in a follow-up survey, and among minority respondents, consistent reporting of race ranged between 55% and 78% [63]. Similar racial fluidity was reported among people identifying as American Indian, Pacific Islander, and multiracial between the 2000 and 2010 US Census. The highest rate of response changes occurred among double-minority groups; for example, among non-Hispanic individuals reporting multiracial American Indian and Pacific Islander background in 2000, only 10% selected the same two categories in 2010 [58].

Several factors that influence the reliability of race and ethnicity reporting over time have been identified at the individual, societal, and study level. First, an individual’s ethnic and racial identity may evolve over time and space–that is, across life stages and social contexts. A “simplification” phenomenon has been described among people of mixed heritage, whereby a multiracial individual may simplify their background into a single race or ethnicity identity when they marry or leave their childhood home [60, 64].

The context in which the question is asked may also affect an individual’s response. An individual may disclose different backgrounds while at home, at work, and during travel, depending on comfort, perceived safety, or benefit of disclosure [53, 58, 60, 62, 65]. In the 1994–1995 US National Longitudinal Study of Adolescent Health, for example, 12% of adolescents reported a different race at home versus at school [66]. In addition to the phenomenon of “passing,” a “chameleon change” experience has been described among multiracial individuals who preferentially identify with different racial groups at different times [67]. Finally, there is increasing concern among Indigenous communities regarding “ethnic fraud” whereby false self-identification of Indigenous heritage is committed by non-Indigenous people in order to gain resources or opportunities [68].

Lastly, study design may influence the reliability of race and ethnicity data. The mode of data collection, such as written surveys or face-to-face interviews, may also yield differing responses [60]. Other factors influencing responses include question wording, response choices, and the instructions and examples available to respondents [50, 53, 60]. Finally, changes in the classification and language of racial and ethnic categories–such as the option of reporting mixed races and ethnicities introduced on the 2000 US and 2001 United Kingdom censuses–may change an individual’s classification over time [54, 56, 62].

*Confusion and conflation of terms*. We have described how race and ethnicity are fluid concepts, and while various definitions for both have been proposed, there is no standard agreement on these definitions. Efforts to collect race- and ethnicity-based data should be iterative, taking caution not to assume these variables are fixed [53]. Interpretation of research findings based on race and ethnicity data must also ensure that results can be appropriately generalized over time [26]. The confusion about the use of these terms has led some researchers to altogether avoid defining race and ethnicity as distinct categories, and instead adopt “race/ethnicity” as a category [69]. Trends in the bibliographic database, MEDLINE, show that researchers are using the term “ethnicity” or “race/ethnicity” instead of “race” more often [70]. While some argue that “race/ethnicity” should be the preferred phrase among researchers, others claim that this categorization is still imprecise, since there are no universally accepted definitions for either concept. As well, while “race/ethnicity” is broader, its use is limited for groups and individuals who do not fall under traditional categories, such as Hispanic populations [69]. The conflation of the terms race and ethnicity can also lead to the misinterpretation that race and ethnicity findings have a biological explanation, without considering the social determinants that usually explain between group differences. Another source of confusion comes from the lack of guidance from gatekeepers of research scholarship, including granting agencies and journal editors regarding which terminology should be used (e.g. race, ethnicity, or ancestry) for the health and biomedical research studies they fund or publish. While journal instructions for authors often advocate for including diverse populations in clinical studies, guidelines are less clear about specific race and ethnicity descriptors [1]. Recently, however, the Journal of the American Medical Association (JAMA) created guidelines on reporting race and ethnicity, noting that “terminology, usage, and word choice are critically important, especially when describing people and when discussing race and ethnicity” [17]. JAMA recommends that authors use specific racial and ethnic classifications rather than collective terms [17] and suggests avoiding the “race/ethnicity” classification, and instead recommending using “race and ethnicity.” This terminology recognizes the diverse populations that exist within racial and ethnic groups [17]. A requirement for precise definitions by granting agencies and journals may also engage researchers to reflect more deeply on these constructs and what roles they play in health research. As societal understandings of race and ethnicity continue to evolve, the use and application of these terms must continue to be reassessed regularly [17].

### 3. Genetically-inferred ethnicity and ancestry

**Fig 1:**
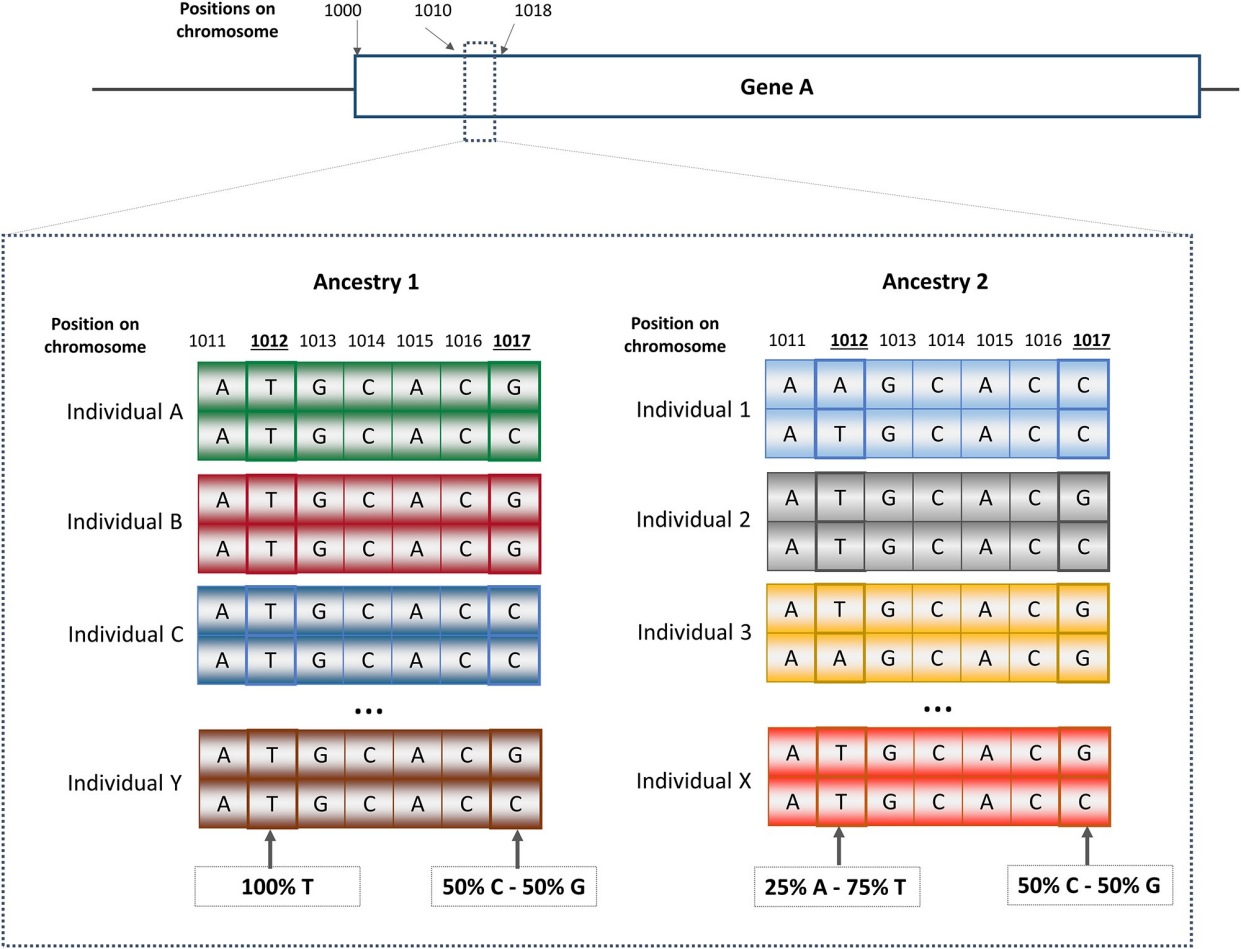
Variation involving 0.1% of the human genome. (A) Proportion of human genome sequence that is similar (blue) and varies (orange) across individuals. (B) A close examination of the DNA sequence (between positions 11 and 17 included) of gene A reveals 2 genetic variants: Variant #1 (position 1012 on chromosome): All participants from Ancestry 1 have a **T** allele, while participants of ancestry 2 have both **T** and **A** allele. This variant could be ethnic specific and could potentially be used as an ancestry marker; Variant #2, (position 1017): the two alleles **G** and **C** are observable in both ancestry groups with similar frequencies, hence, this genetic variant is not an ancestry informative.

Ancestry is another way to characterize individuals beyond race or ethnicity. Ancestry can be defined geographically, genealogically, or genetically, and can suffer limitations similar to race or ethnicity. Geographic ancestry refers to ancestors originating from similar geographic regions. Genealogical ancestry refers to one’s ancestral pedigree, and genetic ancestry refers to ancestors from whom one is biologically descended. It is commonly understood that humans from different ethnic groups are more similar genetically than different; so why is research of genetic differences between diverse populations even undertaken [10–14, 71]? Although the human genome of people from different racial, ethnic, or ancestral groups is ~99.9% identical, some variation is present in a typical human genome compared to a reference genome at 3.7 to 5 million sites representing 0.1% of the entire genome (Fig 1). The vast majority of these genetic variants belong to a class referred to as single nucleotide variants [72, 73], which can be used to determine genetically-inferred ethnicity and/or ancestry. This is because the number of varying sites differs greatly between genetically distant populations (i.e. ~86% of variants are unique to a single ethnic continental group) [72], with populations of African descent typically displaying the largest number of polymorphic sites [72, 73]. In addition, genetic variants that are shared across different ancestral groups also contribute to population differentiation by displaying large differences in allele frequencies. These differences reflect the history and movement of human populations. Although the ancestry of human populations is a continuum, current genetic studies typically stratify populations at the continental level. The term genetic ancestry refers to the outcome of tracing back the DNA of contemporary populations to extinct common ancestors. This is typically done either by comparing contemporary to ancient DNA samples, or by using complex mathematical models.

**Fig 2:**
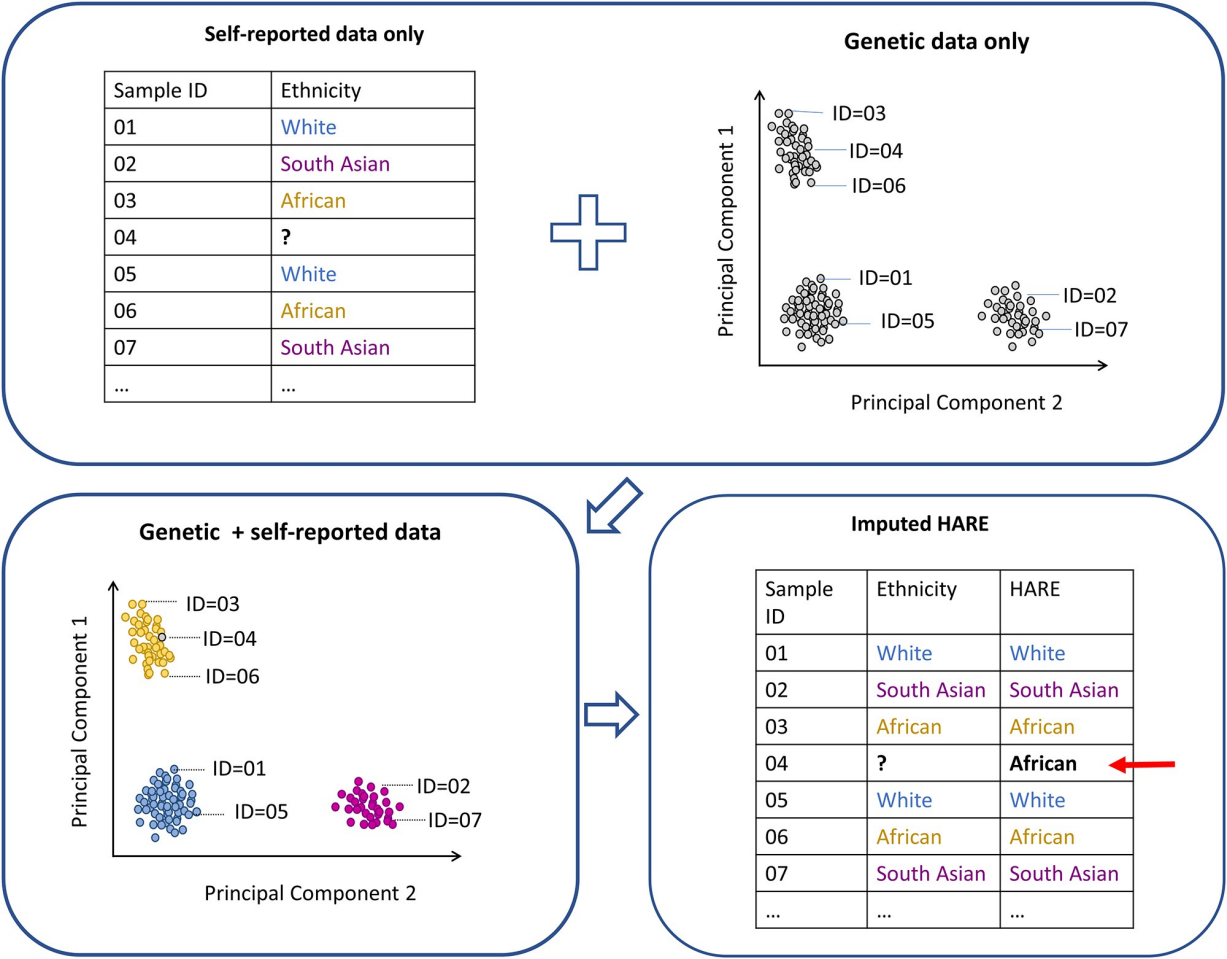
Validation and imputation of self-reported ethnicity using genetic data.

Multiple programs can be used to determine the genetically-inferred ancestry of study participants. These programs can accommodate different study designs and population types (i.e. unrelated [74] vs. related participants [75]; homogenous vs. admixed populations [76, 77]. Among the most commonly used methods are: *i)* Principal component analysis (PCA) [74], a multi-dimensional scaling method implemented in multiple software [75, 78, 79]; and *ii)* model-based clustering approaches [76, 80] used to characterize and visualize genetic ancestry. Genetic ancestry is typically combined with self-reported race or ethnicity data for validation and *vice versa*. In the absence of self-reported data, the information can be inferred using genetic ancestry. This requires the use of samples with validated/harmonized genetic and self-reported data as a reference (Fig 2). A similar approach can also be used to correct or remove data for problematic samples where self-reported race or ethnicity is deemed to be either inaccurate or inconsistent (across time or multiple sources). In an attempt to automate this process of imputation and correction in a large multi-ethnic longitudinal study, a program that combines genetic and self-reported data from multiple sources to generate a “harmonized ancestry and race/ethnicity” variable was recently developed using a machine learning approach [81].

The availability of large reference populations characterizing the genetic structure of non-European populations plays a key role in the accurate inference/validation of genetic ancestry in multi-ethnic and non-European populations, especially when working at a finer scale than the continental level (regions or subregions). However, there has been a lag in whole-genome sequencing and genotyping studies funded, conducted, and published in non-White populations. This has hindered both the ability to refine currently known GWAS signals and the chances of discovering new associations, hence limiting our understanding of the biological mechanisms involved. For example, a recent study revealed massive disparities in predicting accurate genetic risk scores when applying European-derived scores to non-European ancestry populations in UK Biobank, the largest biobank available in the field [82]. A growing number of non-European and multi-ethnic studies and biobanks with available whole-genome/exome sequencing projects have emerged in the last decade to overcome the above limitations, including the 1000Genomes project [72], TOPmed program [73], African Pan-Genome [83], GenomeAsia 100K Project [84], and Singapore WGS project [85, 86]. Large databases aggregating data from multiple sources and ethnic groups are also being developed [87]. However, more efforts are needed to increase diversity and representation in genetic studies.

#### Validity of genetic ethnicity and ancestry data

Contrary to self-reported ethnicity, genetically-inferred ethnicity/ancestry can provide a biologically-based and measurable estimate of an individual’s ancestry. Genetically-inferred ethnicity/ancestry can also provide crucial information that may not be available from self-reported ethnicity, especially among individuals with unknown family ancestry. This information can be informative in genetic investigations, but far less so in non-genetic research. An analysis of genetic studies recording self-reported ethnicity and a harmonized definition of self-reported and genetically-inferred ethnicity concluded that self-reported and genetically-inferred ethnicity have complementary strengths. This variable, termed HARE (harmonized ancestry and race/ethnicity), appears to be a reliable classification method in genetic associations studies [81] and uses genetically-inferred ancestry to refine self-reported race/ethnicity for genetic association studies in three ways: i. to identify individuals whose self-reported race/ethnicity is discordant with genetic ancestry, ii. to reconcile conflicts among multiple sources, and iii. to impute missing racial and ethnic information when the predictive confidence is high. Thus, when an individual’s self-reported ethnicity is ambiguous, either because there are no data or inconsistent responses from multiple sources, genetic information can identify the stratum that most resembles the individual with respect to genetic ancestry as a surrogate for self-reported race or ethnicity [81]. This may be adequate for primarily genetic studies but is not recommended for social sciences and other health studies. For example, an individual living in the US with mixed ancestry for whom self-reported race or ethnicity is missing may be classified as primarily African origin by imputing their genetically-inferred ancestry. If a database combining genetic, social, and health data used genetically-inferred ancestry as a surrogate for self-reported race and ethnicity, then this individual may be grouped with others of self-reported African origin, although this may be completely erroneous, and add substantial noise to social and health-based research questions.

In 2017, a survey of clinical genetics professionals and researchers revealed that a lack of definitions of race, ethnicity, and ancestry in medical research may contribute to inconsistencies in data collection, missing or inaccurate classifications, and misleading or inconclusive results. The authors called for standardization and harmonization of race/ethnicity/ancestry data collection in clinical genetics and precision medicine research–referring to how race, ethnicity, and ancestry are perceived, defined, and measured [88].

### 4. Going forward: Suggestions to improve choice and reporting of terms

**Fig 3:**
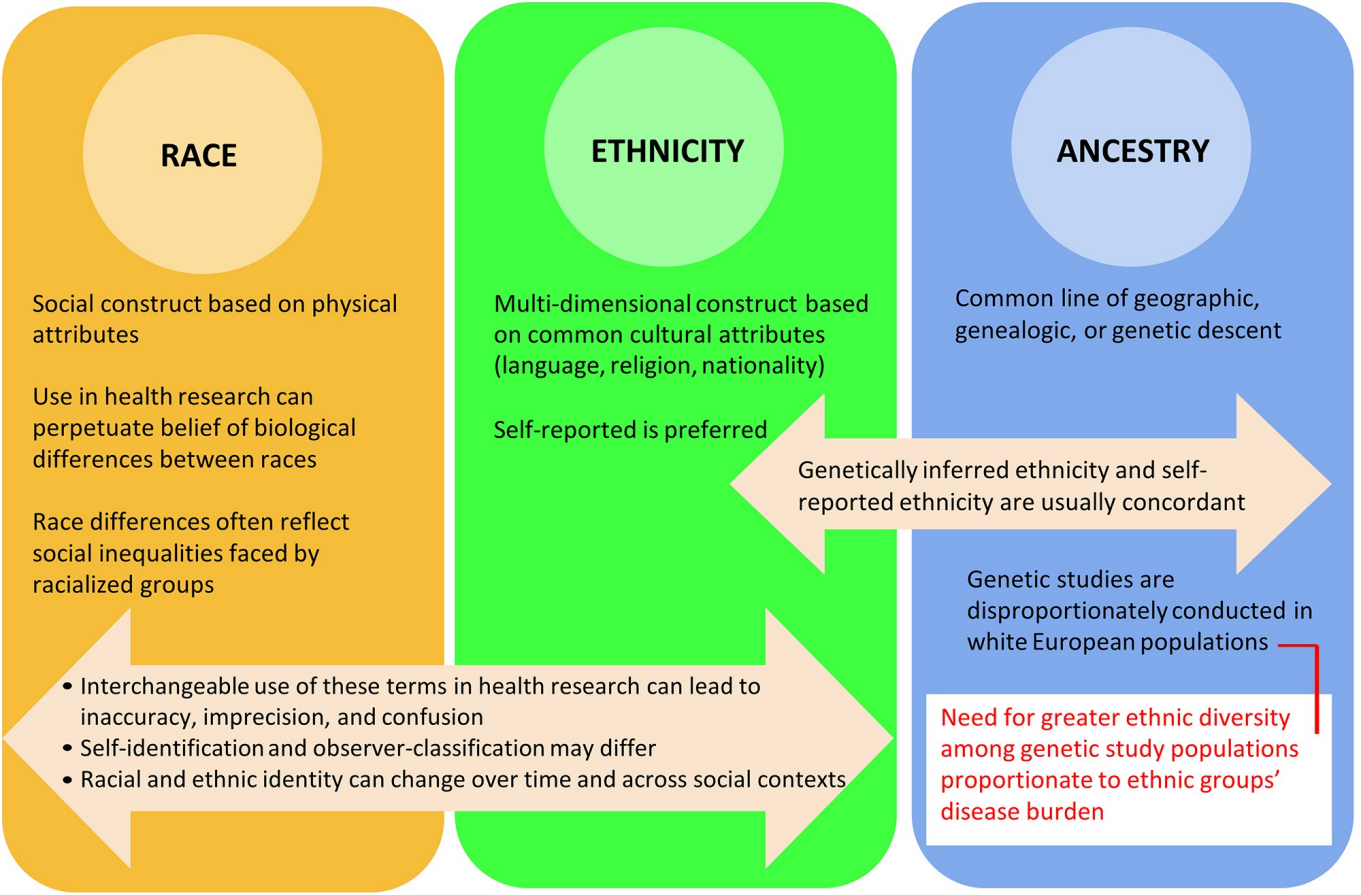
Race, ethnicity, and ancestry considerations in health research.

In 2022, it is clear that the terms race, ethnicity, and ancestry are distinct but overlapping concepts that imperfectly relate to social factors, physical features, cultural characteristics, and ancestral backgrounds. Fig 3 depicts these interrelated concepts. Race is a social construct carrying historical divisions made purely on the basis of physical characteristics, while ethnicity describes a shared cultural background which may include nationality, language, religion, and sometimes common biological characteristics. Ancestry refers to lineage and can refer to common geographic origin, genealogic, or genetic characteristics. Table 1 provides examples of research questions focused on type 2 diabetes mellitus for which using the variables of race, ethnicity, or ancestry may be most appropriate. Although race and ethnicity data are more reliable when self-reported by an individual, even self-reported identities can change over time depending on the individual’s affiliation with their heritage community, perceptions of acceptance, advantage or disadvantage, and concerns for safety. Genetic researchers may use self-reported and/or genetically-inferred ethnicity in scenarios which best suit their research questions, but should be cautious about using genetically-inferred ethnicity alone (especially when investigating gene-environment interactions) or as a proxy for self-reported ethnicity (e.g. when addressing a social science or health services research question) [89]. Observer classification has potential harms such as misclassification, stigmatization, and perpetuating structural racism, but also potential applications–through ethnicity inferences using unique surname classification and through use of discordance between self-report and observer classification–to explore the impacts of biases in healthcare delivery. Considering all of these factors, in order to improve choice of terms and consistency of reporting, we recommend that health researchers: 1) carefully consider the objectives of the research and choose the most appropriate term (i.e. ethnicity, race, or ancestry) and use it consistently; 2) collect self-reported ethnicity, race, or ancestry as preferred over observer-assigned; 3) ask for self reported ethnicity, race or ancestry at each assessment in prospective studies; 4) use self-reported ethnicity together with genetically-inferred ancestry in genetic association studies, and 5) frame data in a manner that maximizes the potential benefit to their study participants, and carefully consider if harm could occur through stigmatization or by perpetuating racism.

**Table 1:** Examples of type 2 diabetes mellitus (T2DM) questions and suggested classification/terms.

	Research question	Suggested Classification/Term*	Rationale
1	Among patients with T2DM, do referrals to diabetic care clinics differ by race?	Race	Implicit bias and structural racism may influence referral patterns.
2	What foods are associated with T2DM in a multicultural population?	Ethnicity	Ethnic groups share common cultural characteristics such as common foods and meal preparation techniques.
3	Does GLP-1 receptor agonist effectiveness in T2DM differ by ethnicity?	Ethnicity	Since race is a social and not a biological construct, ethnicity is preferred.
4	Do genetic variants for T2DM differ by ethnicity?	Ethnicity and genetically-inferred ancestry	Ethnicity and genetically inferred ancestry can be used together to compare ethnic groups.

*For all variables, self-report is preferred over observer classification.

T2DM, type 2 diabetes mellitus; GLP-1, glucagon-like peptide-1.

## Conclusion

There is no consensus definition of race or ethnicity. Every decision to collect race, ethnicity, or ancestry data in the context of health research must involve specific definitions of these terms, justification for collecting and analyzing these data, and wherever possible, prioritization of self-report over observer classification. As our understanding of these concepts evolves, their ongoing refinement is of integral importance: when used appropriately in health research, their collection and analysis can provide invaluable information for health researchers and health equity advocates.

## Supporting information

S1 Table Search strategies. (DOCX)
