# Supplementary material for: Use of race, ethnicity, and ancestry data in health research
Source: PLOS Glob Public Health. 2022 Sep 15;2(9):e0001060. doi: 10.1371/journal.pgph.0001060 (PMC10022242; doi:10.1371/journal.pgph.0001060)
Supplement: S1 Table — (DOCX) [file pgph.0001060.s001.docx]

**Appendix**: Search Strategy by Section

All terms searched were restricted to Title or Abstract. If > 3000 results were found, then MeSH terms were used to restrict results.

| Section | Final Search Strategy | Database | Number of Results | N References from search results |
| --- | --- | --- | --- | --- |
| Ethnicity, Race, Health Research | ("race"[Title/Abstract] OR "racial*"[Title/Abstract] OR "racism"[Title/Abstract] OR "ethnic*"[Title/Abstract]) AND ("health research"[Title/Abstract] OR "health services research"[Title/Abstract] OR "biomedical"[Title/Abstract] OR "epidemiol*"[Title/Abstract] OR "scien*"[Title/Abstract]) AND ("ethnicity"[MeSH Major Topic] OR "racial groups"[MeSH Major Topic] OR "racism"[MeSH Major Topic]) AND ("healthcare disparities"[MeSH Major Topic] OR "health services research"[MeSH Major Topic] OR "biomedical research"[MeSH Major Topic] OR "epidemiologic methods"[MeSH Major Topic] OR "epidemiologic factors"[MeSH Major Topic]) | PubMed | 1173 | 10 |
| Reliability over time | ("race"[Title/Abstract] OR "racial*"[Title/Abstract] OR "ethnic*"[Title/Abstract]) AND ("self-report*"[Title/Abstract] OR "identi*"[Title/Abstract]) AND ("reliab*"[Title/Abstract] OR "evol*"[Title/Abstract] OR "consisten*"[Title/Abstract] OR "chang*"[Title/Abstract] OR "over time"[Title/Abstract] OR "with time"[Title/Abstract] OR "agree*"[Title/Abstract]) AND ("ethnicity"[MeSH Terms] OR "racial groups"[MeSH Terms]) | PubMed | 5677 | 7 |
| Self vs Observer Classification | "self*"[Title/Abstract] AND ("observer*"[Title/Abstract] OR "interviewer*"[Title/Abstract] OR "administ*"[Title/Abstract]) AND ("race*"[Title/Abstract] OR "racial*"[Title/Abstract] OR "ethnic*"[Title/Abstract]) AND ("ethnicity"[MeSH Terms] OR "racial groups"[MeSH Terms] OR "health status disparities"[MeSH Terms] OR "racism"[MeSH Terms] OR "self report"[MeSH Terms]) | PubMed | 1132 | 8 |
| Genetically-Inferred Ethnicity and Ancestry | ("race"[Title/Abstract] OR "racial*"[Title/Abstract] OR "racism"[Title/Abstract] OR "ethnic*"[Title/Abstract] OR "ancest*"[Title/Abstract] OR "racism"[MeSH Terms]) AND ("genetic ancestr*"[Title/Abstract] OR "genetic variation"[Title/Abstract] OR "genetic research"[Title/Abstract]) AND ("ethnicity/genetics"[MeSH Terms] OR "racial groups/genetics"[MeSH Terms] OR "whites/genetics"[MeSH Terms] OR "asians/genetics"[MeSH Terms] OR "blacks/genetics"[MeSH Terms] OR "human/genetics"[MeSH Terms] OR "genetics, population"[MeSH Terms] OR "genetic variation/genetics"[MeSH Terms]) | PubMed | 1961 | 9 |
| Ethnic religions | ("hindu*"[Title/Abstract] OR "judaism*"[Title/Abstract] OR "jew"[Title/Abstract] OR "jewish"[Title/Abstract] OR "amish"[Title/Abstract] OR "mennonite"[Title/Abstract] OR "folk religion"[Title/Abstract] OR "indigenous religion"[Title/Abstract]) AND ("health research"[Title/Abstract] OR "health services research"[Title/Abstract] OR "health evidence"[Title/Abstract] OR "biomedical"[Title/Abstract] OR "epidemiol*"[Title/Abstract] OR "scien*"[Title/Abstract]) | PubMed | 1216 | 1 |
